# Labor market marginalization in individuals with bipolar disorder: a Swedish nationwide register-based sibling comparison study

**DOI:** 10.1017/S0033291724002903

**Published:** 2024-12

**Authors:** Bergný Ármannsdóttir, Heidi Taipale, Aemal Akhtar, Alexander Kautzky, Emma Björkenstam, Johannes Lieslehto, Jari Tiihonen, Ridwanul Amin, Ellenor Mittendorfer-Rutz

**Affiliations:** 1 Department of Clinical Neuroscience, Division of Insurance Medicine, Karolinska Institutet, Stockholm, Sweden; 2 Department of Forensic Psychiatry, University of Eastern Finland, Niuvanniemi Hospital, Kuopio, Finland; 3 School of Pharmacy, University of Eastern Finland, Kuopio, Finland; 4 Center for Psychiatry Research, Stockholm City Council, Stockholm, Sweden

**Keywords:** disability pension, mood disorders, severe mental illness, sick leave, unemployment, work disability

## Abstract

**Background:**

There is a lack of large-scale studies exploring labor market marginalization (LMM) among individuals diagnosed with bipolar disorder (BD). We aimed to investigate the association of BD with subsequent LMM in Sweden, and the effect of sex on LMM in BD.

**Methods:**

Individuals aged 19–60 years living in Sweden with a first-time BD diagnosis between 2007 and 2016 (*n* = 25 231) were followed from the date of diagnosis for a maximum of 14 years. Risk of disability pension (DP), long-term sickness absence (SA) (>90 days), and long-term unemployment (>180 days) was compared to a matched comparison group from the general population, matched 1:5 on sex and birth year (*n* = 126 155), and unaffected full siblings (*n* = 24 098), using sex-stratified Cox regression analysis, yielding hazard ratios (HRs) with 95% confidence intervals (CIs).

**Results:**

After adjusting for socioeconomic factors, baseline labor market status, and comorbid disorders, individuals with BD had a significantly higher risk of DP compared to the general population (HR = 16.67, 95% CI 15.33–18.13) and their unaffected siblings (HR = 5.54, 95% CI 4.96–6.18). Individuals with BD were also more likely to experience long-term SA compared to the general population (HR = 3.19, 95% CI 3.09–3.30) and their unaffected siblings (HR = 2.83, 95% CI 2.70–2.97). Moreover, individuals diagnosed with BD had an elevated risk of long-term unemployment relative to both comparison groups (HR range: 1.75–1.78). Men with BD had a higher relative risk of SA and unemployment than women. No difference was found in DP.

**Conclusions:**

Individuals with BD face elevated risks of LMM compared to both the general population and unaffected siblings.

## Introduction

Bipolar disorder (BD) is a chronic and severe mood disorder, affecting approximately 1–3% of the global population (Vieta et al., 2018). BD is one of the leading causes of disability worldwide, with up to 60 million people affected (Vigo, Thornicroft, & Atun, 2016). The Global Burden of Disease Study estimated that BD resulted in 8.5 million disability-adjusted life-years among people living with the disorder globally in 2019 (GBD 2019 Mental Disorders Collaborators, 2022).

Individuals with BD endure episodes of intense mood fluctuations that can significantly disrupt their daily lives and functioning (Michalak, Yatham, Maxwell, Hale, & Lam, 2007; Vieta et al., 2018). BD often impairs normal functioning of the individual significantly across multiple dimensions, even during periods of remission (Michalak et al., 2007). Social inclusion, such as participation in the labor market, is associated with positive outcomes for individuals with BD (Baumgartner & Susser, 2013), but is challenged by the considerable restrictions in occupational functioning caused by the disease (Huxley & Thornicroft, 2003).

Employment rates among individuals with BD have consistently been reported to range between 40% and 60% (Arvilommi et al., 2022; Dominiak et al., 2022; Hakulinen et al., 2020; Hakulinen, Musliner, & Agerbo, 2019; Kupfer et al., 2002; Marwaha, Durrani, & Singh, 2013). Swedish register-based studies revealed that only 40% of individuals diagnosed with BD in Sweden were gainfully employed, and those who were employed experienced higher levels of sickness absence (SA) compared to those without BD (Carlborg, Ferntoft, Thuresson, & Bodegard, 2015; Holm, Taipale, Tanskanen, Tiihonen, & Mitterdorfer-Rutz, 2021). Lower rates of gainful employment contrasted by increased SA, unemployment, and early exit from the workforce due to disability indicate labor market marginalization (LMM). Previous research has shown that using only one measure for LMM might underestimate the effects mental illness has on work capacity (Helgesson, Tinghög, Niederkrotenthaler, Saboonchi, & Mittendorfer-Rutz, 2017). Therefore, in this study, we seek to move beyond the more traditional binary assessment of employment status and examine LMM by looking at three different labor market outcomes: long-term unemployment, long-term SA, and disability pension (DP).

Large-scale studies that assess more than one labor market outcome for people with BD are scarce. Furthermore, to our knowledge, only one other study has studied siblings of people diagnosed with BD to attempt controlling for unmeasured familial factors that might affect socioeconomic outcomes in people with BD. That particular study, conducted in Denmark, found that individuals with BD exhibited lower rates of employment and educational attainment compared to a matched comparison group from the general population. However, the study also revealed that unaffected siblings of those with BD had worse socioeconomic outcomes when compared to the general population (Sletved, Ziersen, Andersen, Vinberg, & Kessing, 2021). These findings highlight the potential influence of genetics or psychosocial and socioeconomic factors in upbringing on socioeconomic and LMM outcomes in adulthood.

In our study we employed a sibling comparison design, in addition to examining three distinct labor market outcomes, to hopefully provide a more comprehensive understanding of LMM among individuals diagnosed with BD. The sibling comparison method allows us to control for factors such as common genetics as well as psychosocial and socioeconomic factors experienced during childhood and adolescence – collectively referred to as familial factors – that are difficult to measure or quantify. By employing this method, we hope to gain valuable insights into the specific contributions of these familial factors to LMM among individuals with BD.

Previous studies have indicated that certain factors correlate with adverse labor market outcomes among people diagnosed with BD. For example, individuals with BD often experience psychiatric and somatic comorbidities, which have been shown to significantly impact their socioeconomic status and employment prospects (Arvilommi et al., 2022; Gilbert & Marwaha, 2013; Grande et al., 2013; Krishnan, 2005; McIntyre et al., 2006). Regarding sex, a previous study on individuals living with BD in Sweden showed that men with BD have a higher rate of employment than women (Holm et al., 2021). In general, women tend to be more likely to be on DP and long-term SA than men, especially SA due to mental disorders (Gjesdal, Ringdal, Haug, & Gunnar Mæland, 2008; Haukenes, Gjesdal, Rortveit, Riise, & Mæland, 2012; Laaksonen et al., 2010). Therefore, there is reason to suspect that women might have a higher risk of adverse labor market outcomes than men among those diagnosed with BD. However, longitudinal research is scarce, and few large-scale population-based studies have been published to date.

The primary aim of this population-based longitudinal register study was to (1) assess to which extent people with BD have a higher risk of LMM measured as long-term unemployment, long-term SA, and DP, compared to (i) the general population and (ii) their unaffected siblings. Furthermore, a secondary aim was (2) to investigate the role of sex in influencing the subsequent risk of LMM among individuals with BD.

## Methods

### Registers

Information from five population-based registers was linked together using a de-identified unique personal identity number, assigned to each Swedish citizen or resident. The following three are held by the National Board of Health and Welfare:

(1) *The National Patient Register* (NPR), which has information on everyone receiving care in psychiatric or general hospitals, with extensive coverage for inpatient care since 1987 and specialized outpatient care since 2001 (Ludvigsson et al., 2011). Information on main and secondary diagnosis was obtained from the NPR. (2) *The Cause of Death Register* comprises information on all deaths occurring in Sweden since 1952 (Brooke et al., 2017). (3) *The Prescribed Drug Register* (PDR) contains information on dispensed prescribed drugs to the entire Swedish population since July 2005 (Wettermark et al., 2007). Pharmaceuticals in PDR are classified using the Anatomical Therapeutic Chemical (ATC) system.

Additionally, information on sociodemographic factors, as well as data from the labor market and from the educational and social sectors was obtained through *The Longitudinal Integration Database for Health Insurance and Labour Market Studies*, held by Statistics Sweden since 1985 (Ludvigsson, Svedberg, Olen, Bruze, & Neovius, 2019). Information on start and end dates and grade of SA and DP as well as corresponding diagnoses was obtained through the *Microdata for Analyses of Social Insurance* register, held by the Social Insurance Agency (Österlund, 2011).

### Study population

**Figure 1.**
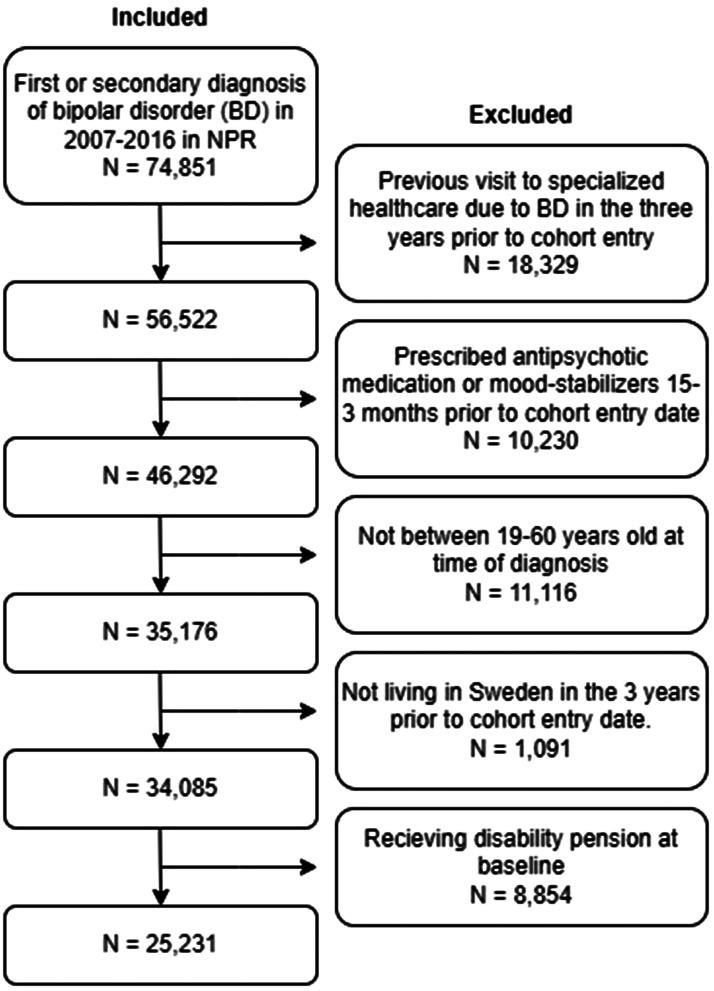
Selection of people with BD included in the study.

We identified 74 851 individuals in the NPR with a first or secondary diagnosis of BD or manic episode (International Classification of Diseases, 10th Revision [ICD-10] codes F30 and F31) between 2007 and 2016 from NPR. The cohort entry date (CED) was determined by the date of their index diagnosis. To select individuals with an incident diagnosis of BD, those who had a previous visit in specialized healthcare within 3 years before cohort entry due to BD (ICD-10 F30, F31), schizophrenia-spectrum disorders (ICD-10 F20–29), or dementia (ICD-10 F00–F03 and G30) were excluded from the study (*n* = 18 329). For similar reasons, individuals who had been prescribed antipsychotic medication (ATC code N05A) or mood-stabilizers (carbamazepine N03AF01, valproic acid N03AG01, lamotrigine N03AX09, lithium N05AN01) in the 15 to 3 months before their diagnosis were also excluded from the study (*n* = 10 230). Additionally, anyone under the age of 19 or over 60 were excluded (*n* = 11 062). The minimum age for DP eligibility in Sweden is 19, and individuals aged 60 or older were excluded to ensure a better chance of capturing those with at least 4 more years of potential participation in the labor market before opting for old age pension. Further exclusions were made for individuals who had not resided in Sweden during the 3 years prior to CED (*n* = 1191) and those who were receiving ongoing DP upon cohort entry (*n* = 8854). Consequently, the final study population comprised of 25 231 individuals (Fig. 1).

Two comparison groups were defined from the same registers. First a matched comparison group (1:5 matching on sex and birth year) of individuals without a BD diagnosis in specialized health care during 2007–2016 from the general population, that did not have an ongoing DP and did not have a diagnosis of schizophrenia-spectrum disorders or dementia. Matching for the individuals in the comparison group was on the CED of the individual with BD. In total, there were 126 155 controls in the matched comparison group.

The other comparison group included full siblings, aged 19–60, without a BD diagnosis and without schizophrenia-spectrum disorders or dementia. We identified 15 769 individuals in the BD group that had at least one full sibling, not affected by BD during 2007–2016. In case of two (or more) siblings with BD, the sibling with the earlier CED was chosen as the exposed individual. In total, we identified 24 098 unaffected full siblings.

### Swedish social insurance system

Individuals aged 16 or older in Sweden, who are unemployed, are entitled to unemployment benefits provided by the Swedish Public Employment Services. Those above 20 years old can receive these benefits without prior work experience. Regarding SA, any resident of Sweden aged 16 or above with a confirmed income can claim sickness benefits in case of restricted work capacity due to disease or injury. During the first 14 days of SA, excluding the first day, the employer is usually responsible for covering the costs for their employees. After the first 14 days the Swedish Social Insurance Agency takes over the payments, and the data are recorded in the national registers from that point forward. For individuals experiencing reduced work capacity, temporary DP is available for those aged 19–29, which can also be granted in the case that compulsory education is not completed. However, upon reaching 30 years, only permanent DP is granted.

### Labor market outcomes

The outcome measures analyzed were (1) DP, (2) long-term SA, and (3) long-term unemployment. In addition, (4) a combined LMM measure was analyzed, defined as experiencing any of the three outcomes. Long-term SA was defined as 90 or more consecutive net days of SA, regardless of grade (i.e. 25%, 50%, 75%, or full-time SA). Only those who were in gainful employment in the calendar year before the cohort entry year (CEY) were included in the long-term SA analysis. Long-term unemployment was defined as 180 or more annual days of unemployment during the follow-up time.

For DP and long-term SA, the actual starting dates were used to determine the event date. For long-term unemployment, the date of the event was set as January 1st of the initial calendar year with more than 180 annual days of unemployment benefit.

### Covariates

Covariates of interest included age, sex, educational level, family composition, type of living area, and global region of birth (see Table 1 for categorization). Those were all measured on December 31st, the year before each individual's cohort entry. Work-related factors were baseline unemployment and SA, also measured the year before cohort entry, as annual net days. Additionally, information on comorbid disorders, based on main and secondary diagnoses of both inpatient and specialized outpatient care in the 3 years before cohort entry for each individual, was included. The following disorders were included: alcohol use disorder (ICD-10 F10), other substance use disorders (F11–F19), depressive disorders (F32–F33), anxiety- and stress-related disorders (F40–48), personality disorders (F60–F69), attention deficit-hyperactivity disorder (ADHD; F90), autism spectrum disorder (F84), disorders of the nervous system (G00–G99, excluding G30), other psychiatric disorders (all remaining ICD-10 F codes), neoplasms (C00-D48), circulatory disorders (I00–I99), musculoskeletal disorders (M00–99), diabetes (E10–E14), and history of suicide attempt (X60–84 and Y10–34). For diabetes, information on antidiabetic medications (ATC code A10) was also used. All categorizations of the covariates are provided in Table 1.

**Table 1.** Characteristics of individuals with BD, diagnosed in specialized health care in 2007–2016, individuals without BD (general population, matched for sex and birth year), and the unaffected full siblings of the individuals with BD

Variable	Individuals with BD	Matched comparison group	Unaffected Siblings
*N*	25 231	126 155	24 098
Sex, *n* (%)^a^			
Male	9546 (37.8)	47 730 (37.8)	12 465 (51.7)
Age, *n* (%)			
19–24 years	5325 (21.1)	26 625 (21.1)	4296 (17.8)
25–34 years	7983 (31.6)	39 915 (31.6)	7913 (32.8)
35–44 years	6269 (24.9)	31 345 (24.9)	6025 (25.0)
45–54 years	4256 (16.9)	21 280 (16.9)	4513 (18.7)
55–64 years	1398 (5.5)	6990 (5.5)	1351 (5.6)
Educational level, *n* (%)^a^^,^^b^			
Low (<10 years)	4796 (19.0)	17 880 (14.2)	3333 (13.8)
Medium (10–12 years)	12 339 (48.9)	59 265 (47.0)	12 048 (50.0)
High (>12 years)	8096 (32.1)	49 010 (38.9)	8717 (36.2)
Type of living area, *n* (%)^b^			
Big cities (Stockholm, Gothenburg, or Malmö)	9637 (38.2)	53 977 (42.8)	9278 (38.5)
Medium-sized cities (>90 000 inhabitants)	10 649 (42.2)	50 027 (39.7)	9943 (41.3)
Small cities/villages (⩽90 000 inhabitants)	4945 (19.6)	22 151 (17.6)	4877 (20.2)
Family composition, year before CEY *n* (%)^a^^,^^b^			
Married/cohabiting without children	1674 (6.6)	11 964 (9.5)	2279 (9.5)
Married/cohabiting with children^c^	5866 (23.3)	42 260 (33.5)	7905 (32.8)
Single/divorced/widowed without children	15 003 (59.5)	64 974 (51.5)	12 536 (52.0)
Single/divorced/widowed with children^c^	2688 (10.7)	6957 (5.5)	1378 (5.7)
Global region of birth, *n* (%)^a^^,^^b^			
Sweden	22 352 (88.6)	104 749 (83.0)	22 854 (94.8)
Nordic countries (except for Sweden)	534 (2.1)	2107 (1.7)	359 (1.5)
Europe (except for Nordic countries)	528 (2.1)	3016 (2.4)	98 (0.4)
Outside Europe	1817 (7.2)	16 283 (12.9)	787 (3.3)
Unemployment at baseline, *n* (%)			
Under 180 days^a^^,^^b^	4699 (18.6)	12 888 (10.2)	2651 (11.0)
Over or equal to 180 days^a^^,^^b^	1232 (4.9)	3302 (2.6)	651 (2.7)
Sick leave in the year before CED, *n* (%)			
Under 90 days^a^^,^^b^	3565 (14.1)	7383 (5.9)	1597 (6.6)
Over or equal to 90 days^a^^,^^b^	4701 (18.6)	3519 (2.8)	941 (3.9)
Psychiatric comorbidity in the 3 years before CED, *n* (%)			
Alcohol use^a^^,^^b^	2030 (8.1)	691 (0.6)	285 (1.2)
Other substance use^a^^,^^b^	1408 (5.6)	540 (0.4)	219 (0.9)
Personality disorders^a^^,^^b^	1704 (6.8)	362 (0.3)	155 (0.6)
ADHD^a^^,^^b^	2499 (9.9)	749 (0.6)	314 (1.3)
Depressive disorders^a^^,^^b^	9401 (37.3)	2200 (1.7)	752 (3.1)
Anxiety disorders^a^^,^^b^	7617 (30.2)	2415 (1.9)	800 (3.3)
Stress-related disorders^a^^,^^b^	3170 (12.6)	1320 (1.1)	398 (1.7)
Suicide attempts^a^^,^^b^	643 (2.6)	252 (0.2)	69 (0.3)
Autism spectrum disorders^a^^,^^b^	320 (1.3)	135 (0.1)	62 (0.3)
Other mental disorders^a^^,^^b^	7637 (30.3)	1753 (1.4)	5557 (2.3)
Somatic disorders in the 3 years before CED, *n* (%)			
Neoplasms/cancer^a^	1381 (5.5)	6342 (5.0)	1117 (5.5)
Neurological disorders^a^^,^^b^	1732 (6.9)	3584 (2.8)	756 (3.1)
Circulatory disorders^a^^,^^b^	1046 (4.2)	3521 (2.8)	712 (3.0)
Respiratory disorders^a^^,^^b^	1858 (7.4)	5856 (4.6)	1134 (4.7)
Musculoskeletal disorders^a^^,^^b^	3658 (14.5)	12 012 (9.5)	2521 (10.5)
Diabetes^b^	412 (1.6)	1525 (1.2)	280 (1.2)

a Statistically significant between-group difference (*p* < 0.001) between those diagnosed with BD compared to unaffected full siblings, determined with a χ^2^ test.

b Statistically significant between-group difference (*p* < 0.001) between those diagnosed with BD compared to the matched comparison group from the general population, determined with a χ^2^ test.

c With children living at home.

### Statistical analyses

Chi-squared tests were used to examine statistically significant differences between the groups. Cox proportional hazard regression models were employed to assess the influence of BD on LMM outcomes. Schoenfeld residuals were used to visually assess the proportional hazard assumptions. No deviations over time were observed, indicating acceptable proportional hazards.

We assessed the person-years at risk from date of diagnosis until either the first LMM outcome, emigration, old age pension, death, or end of follow-up (December 31, 2020), whichever took place first. In the analyses concerning long-term SA and long-term unemployment, DP was also treated as a censor. Hazard ratios (HRs) with 95% confidence intervals (CIs) were used to present the results.

We constructed four models for each outcome variable: one unadjusted model and three adjusted models. Each adjusted model built on the previous one, including additional covariates to those already accounted for in the previous model. The first adjusted model incorporated adjustments for socioeconomic variables – i.e. education level, family composition, type of living area, and region of birth. In addition, adjustments for sex and age were incorporated in all models of the sibling comparison, including the unadjusted model, as siblings were not matched on sex and birth year, unlike the matched comparison group. The second adjusted model added adjustment for unemployment and SA at baseline, i.e. in the calendar year before the CEY. The third model additionally adjusted for comorbid mental and somatic disorders. All models were done both for the matched comparison and the sibling comparison. Only those in the BD group that had an unaffected full sibling were included in the sibling analysis.

Sex-stratified Cox models were used to examine HRs for men and women separately. Additionally, a multimodal partial likelihood ratio test was employed to compare differences in HRs between men and women. Data analysis was performed using SAS 9.4.

## Results

The mean age of the individuals with BD, and therefore also the matched comparison group, was 35.0 years (=11.0) and 38% were male. The mean age of the unaffected siblings was 35.7 (s.d. = 10.8) and 52% were male. Median follow-up time was 7.9 years (interquartile range 5.7–10.6).

Individuals diagnosed with BD were more likely than the comparison groups to have any comorbid disorder, both psychiatric and somatic (Table 1). Almost 40% of those with BD had been diagnosed with a depressive disorder in the 3 years prior to cohort entry, compared to under 2% of the matched comparison group and 3% among unaffected siblings (*p* < 0.001). Moreover, 30% of the individuals with BD had been diagnosed with anxiety disorders in the 3 years prior to cohort entry, compared to approximately 2% of the matched comparison group and 3% of the unaffected siblings (*p* < 0.001).

Those diagnosed with BD were also more likely than the matched comparison group to have periods of SA over or equal to 90 days (18.63% *v.* 2.79% respectively, *p* < 0.001), or long-term unemployment, over or equal to 180 days (4.88% *v.* 2.62% respectively, *p* < 0.001) in the year before cohort entry. This was also true when compared to their unaffected siblings, where 2.7% experienced over 180 days of unemployment at baseline and 3.9% had a period of SA absence, in the year before cohort entry. Furthermore, as stated earlier, 8854 individuals diagnosed with BD were already on DP at cohort entry, highlighting the considerable proportion of people with BD who are outside the labor market before receiving their diagnosis, suggesting a substantial burden of comorbidity.

**Figure 2.**
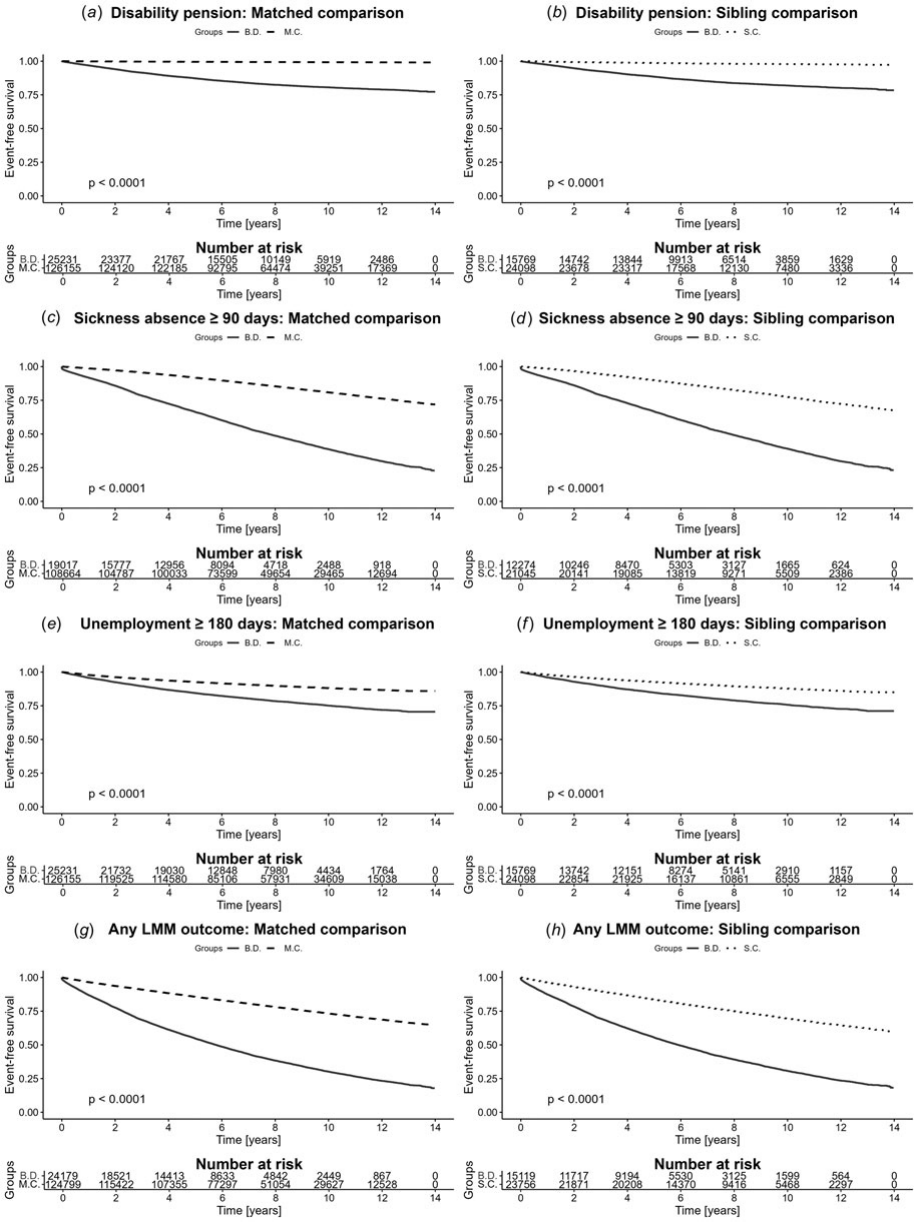
Kaplan–Meier estimates of survival functions for each outcome in the study, for both comparison groups.

During the follow-up period, 16.9% of individuals diagnosed with BD received DP. In contrast, only 0.7% of the matched comparison group (Table 2) and 1.9% of the unaffected siblings (Table 3) received DP. In the most adjusted model, adjusted for socioeconomic factors, labor market status, and comorbid disorders, the HR for the matched comparison group was 16.67 (95% CI 15.33–18.13) (Table 2, Fig. 2a). The HRs reduced somewhat by each adjustment made, the most between models 2 and 3, when adding adjustments for SA and unemployment at baseline (HR = 26.72 *v.* HR = 20.22 respectively). When compared to their unaffected siblings the most adjusted model for DP yielded the HR 5.54 (95% CI 4.96–6.18) (Table 3, Fig. 2b).

**Table 2.** HRs and corresponding 95% CIs for LMM outcomes among individuals aged 19–60 diagnosed with BD in specialist care between 2007 and 2016, compared with unaffected individuals from the general population matched on sex and birth year, stratified by gender

	Individuals with BD (*n* = 25 231)			Matched comparison group (*n* = 126 155)						
	*n*	%	Incidence rate^a^	*n*	%	Incidence rate^a^	Unadjusted model, HR (95% CI)	Adjusted model 1^b^, HR (95% CI)	Adjusted model 2^c^, HR (95% CI)	Adjusted model 3^d^, HR (95% CI)
DP										
All	4251	16.85	6.35	832	0.66	0.22	28.43 (26.40–30.63)	26.72 (24.78–28.81)	20.22 (18.70–21.86)	16.67 (15.33–18.13)
Women	2818	17.97	6.76	544	0.69	0.23	28.80 (26.28–31.57)	26.92 (24.53–29.54)	20.35 (18.47–22.41)	16.70 (15.05–18.53)
Men	1433	15.01	5.68	288	0.60	0.20	27.74 (24.44–31.48)	26.23 (23.09–29.80)	20.03 (17.54–22.87)	16.77 (14.55–19.33)
SA ⩾90 days										
All	11 949	47.36	24.63	19 402	15.38	5.58	4.62 (4.50–4.73)	4.59 (4.47–4.71)	3.59 (3.49–3.69)	3.19 (3.09–3.30)
Women	8011	51.07	25.14	14 336	18.28	6.54	4.14 (4.02–4.27)	4.11 (3.99–4.24)	3.25 (3.14–3.36)	2.88 (2.76–2.99)
Men	3938	41.25	26.47	5066	10.61	3.70	5.89 (5.63–6.16)	5.94 (5.68–6.22)	4.72 (4.49–4.96)	4.27 (4.09–4.53)
Unemployment ⩾180 days										
All	4896	19.40	8.37	12 930	10.25	3.62	2.24 (2.16–2.31)	2.27 (2.19–2.35)	1.94 (1.87–2.01)	1.78 (1.71–1.86)
Women	2677	17.07	7.19	7673	9.78	3.43	2.04 (1.95–2.13)	2.07 (1.98–2.17)	1.80 (1.71–1.88)	1.66 (1.57–1.76)
Men	2219	23.25	10.45	5257	11.01	3.93	2.54 (2.42–2.67)	2.54 (2.42–2.67)	2.12 (2.01–2.23)	1.91 (1.79–2.04)
Any LMM outcome										
All	14 820	61.29	33.26	28 239	22.63	8.50	3.90 (3.82–3.98)	3.88 (3.80–3.96)	3.09 (3.02–3.15)	2.74 (2.67–2.81)
Women	9431	63.11	33.79	19 038	24.62	9.30	3.67 (3.58–3.76)	3.63 (3.54–3.73)	2.91 (2.83–2.99)	2.57 (2.48–2.65)
Men	5389	58.35	32.36	9201	19.38	7.22	4.36 (4.21–4.51)	4.37 (4.22–4.52)	3.48 (3.36–3.61)	3.13 (3.00–3.27)

a Events per 100 000 person-years.

b Adjusted for education level, family composition, type of living area, and region of birth.

c Same as model 1 and additionally adjusted for unemployment and SA at baseline.

d Same as model 2 and additionally adjusted for comorbidities of mental and somatic disorders.

**Table 3.** HRs and corresponding 95% CIs for LMM outcomes among individuals aged 19–60 diagnosed with BD in specialist care between 2007 and 2016, compared with their unaffected full siblings, stratified by gender

	BD individuals with unaffected full siblings (*n* = 15.769)			Unaffected full siblings of BD individuals (*n* = 24.098)			Model adjusted for age and sex, HR (95% CI)	Adjusted model 1^b^, HR (95% CI)	Adjusted model 2^c^, HR (95% CI)	Adjusted model 3^d^ HR (95% CI)
	*n*	%	Incidence rate^a^	*n*	%	Incidence rate^a^				
DP										
All	2471	15.67	5.80	458	1.90	0.63	9.04 (8.18–9.99)	8.64 (7.81–9.55)	6.66 (6.00–7.39)	5.54 (4.96–6.18)
Women	1642	16.84	6.23	272	2.34	0.77	8.44 (7.42–9.60)	8.06 (7.07–9.17)	6.11 (5.35–6.99)	5.13 (4.45–5.91)
Men	829	13.78	5.11	186	1.49	0.50	10.26 (8.75–12.04)	9.76 (8.32–11.45)	7.68 (6.51–9.06)	6.24 (5.23–7.45)
SA ⩾90 days										
All	6284	51.20	24.44	3970	18.86	6.74	3.63 (3.49–3.78)	3.60 (3.45–3.75)	3.08 (2.95–3.22)	2.83 (2.70–2.97)
Women	4160	54.61	26.23	2521	24.77	9.07	3.12 (2.97–3.28)	3.08 (2.93–3.24)	2.64 (2.51–2.79)	2.42 (2.28–2.57)
Men	2124	45.61	21.55	1449	13.34	4.65	4.69 (4.38–5.01)	4.67 (4.36–5.00)	3.96 (3.69–4.26)	3.68 (3.39–3.98)
Unemployment ⩾180 days										
All	3029	19.21	8.12	2531	10.51	3.72	2.22 (2.10–2.34)	2.06 (1.95–2.17)	1.85 (1.74–1.95)	1.75 (1.64–1.87)
Women	1636	16.77	6.94	1099	9.45	3.12	2.04 (1.89–2.20)	1.86 (1.72–2.01)	1.67 (1.53–1.81)	1.59 (1.45–1.74)
Men	1393	23.15	10.16	1432	11.49	4.10	2.41 (2.24–2.59)	2.25 (2.09–2.42)	2.03 (1.88–2.19)	1.91 (1.74–2.08)
Any LMM outcome										
All	9224	61.01	32.57	6175	25.99	9.90	3.25 (3.15–3.36)	3.14 (3.03–3.24)	2.68 (2.59–2.77)	2.45 (2.35–2.54)
Women	5829	62.71	33.14	3427	30.11	11.65	2.97 (2.84–3.10)	2.85 (2.73–2.98)	2.43 (2.32–2.54)	2.20 (2.09–2.32)
Men	3395	58.29	31.64	2748	22.21	8.34	3.70 (3.52–3.89)	3.57 (3.40–3.76)	3.07 (2.91–3.24)	2.83 (2.66–3.01)

a Events per 100 000 person-years.

b Adjusted for education level, family composition, type of living area, and region of birth.

c Same as model 1 and additionally adjusted for unemployment and SA at baseline.

d Same as model 2 and additionally adjusted for comorbidities of mental and somatic disorders.

Among individuals diagnosed with BD, 47.4% experienced long-term SA during the study period, compared to 15.4% of the matched comparison group (Table 2) and 18.9% of the unaffected siblings (Table 3). In the most adjusted model, the HR for long-term SA when comparing those diagnosed with BD to the matched comparison group was 3.19 (95% CI 3.09–3.30) (Table 2, Fig. 2c). Similarly, in the sibling comparison, the most adjusted model yielded an HR of 2.83 (95% CI 2.70–2.97) for long-term SA (Table 3, Fig. 2d). The HRs reduced somewhat with each additional adjustment made in both the sibling comparison (crude HR = 3.63) and the matched comparison (crude HR = 4.62).

The BD and matched comparison group differed less in terms of long-term unemployment (19.4% *v.* 10.3% respectively) over the follow-up time, compared to other LMM outcomes, with an HR of 1.78 (95% CI 1.71–1.86) in the most adjusted model (Table 2, Fig. 2e). Among the unaffected siblings, 10.5% experienced long-term unemployment during the study period. When comparing unaffected siblings to those with BD, the HR was 1.75 (95% CI 1.64–1.87) in the most adjusted model (Table 3, Fig. 2f).

During the study period, 61% of the individuals diagnosed with BD had some form of LMM, compared to 22.6% of the matched comparison group (HR = 2.74 [95% CI 2.67–2.81]) (Table 2, Fig. 2g) and 26% of the unaffected siblings (HR = 2.45 [95% CI 2.35–2.54]) (Table 3, Fig. 2h).

### Sex differences

Both men and women with a BD diagnosis had a higher likelihood of any of the LMM outcomes, compared with men and women from the matched comparison group. Women had a higher absolute risk across all outcomes, but the relative risk associated with BD was higher for men than for women across all outcomes, except for DP (Table 2). The difference in risk was significant between men and women for long-term SA (HR = 2.88 *v.* 4.27, *p* < 0.001), long-term unemployment (HR = 1.66 *v.* 1.91, *p* < 0.001), and for any LMM (HR = 2.57 *v.* 3.13, *p* < 0.001), but there was no significant difference in HRs regarding DP (HR = 16.70 *v.* 16.77, *p* = 0.69).

## Discussion

### Main findings

This study found that during the study period of up to 14 years, over 60% of people diagnosed with BD in Sweden had some form of LMM. This is almost a three-fold increase compared to the matched comparison group from the general population. The risk of being granted DP was almost 17-fold higher than the general population and over five-fold higher than the risk of their unaffected siblings, when controlling for socioeconomic status, comorbidities, and LMM at baseline. People with BD were also around three times more likely than the general population and the unaffected siblings to have periods of long-term SA. Men had had a higher relative risk than women of long-term SA, long-term unemployment, and any LMM outcome.

About 17% of those diagnosed with BD received DP during the follow-up period, compared to 0.7% of the general population and 1.9% of their unaffected siblings. A Danish register study from 2022 found that 20% of people were receiving DP 3 years after a BD diagnosis (Christensen, Wallstrøm, Eplov, Laursen, & Nordentoft, 2022). However, they did not exclude individuals already on DP at the time of diagnosis, which might account for the higher DP frequency despite the shorter follow-up time.

Putting our findings in the context of similar research on labor market outcomes in different psychiatric disorders, the estimated impact of BD is second only to schizophrenia and non-affective psychosis, who had a 25-fold higher risk of receiving DP compared to the general population (Helgesson et al., 2017). People with BD had a similar risk of receiving DP as people diagnosed with obsessive-compulsive disorder (OCD) (16-fold risk compared to the general population) (Perez-Vigil, Mittendorfer-Rutz, Helgesson, Fernandez de la Cruz, & Mataix-Cols, 2019).

Almost 50% of those diagnosed with BD had a period of long-term SA during the follow-up period, compared to 15% of the general population, resulting in a 3.19-fold higher risk for those with BD. In the sibling comparison, the results were similar. Comparing these estimates to other psychiatric disorders puts BD at around the same risk estimate as schizophrenia and non-affective psychosis, where the risk of long-term SA was 3.16-fold compared to the general population, and considerably higher than all other psychiatric disorders (Helgesson et al., 2017).

The HRs for long-term unemployment were lower than the other LMM outcomes, but still there was a 1.78-fold higher risk for those with BD compared to the general population and a 1.75-fold higher risk when compared to unaffected siblings. About 19% of those with BD had a period of long-term unemployment during the follow-up. This is somewhat lower than what other, smaller studies have shown, with some estimating unemployment as high as 60% (Hakulinen et al., 2019; Sletved et al., 2021). However, previous studies tend to treat unemployment as synonymous with being out of the labor market, without differentiating between various LMM outcomes or defining the duration of unemployment (Dominiak et al., 2022; Marwaha et al., 2013), perhaps making comparison unfeasible. When comparing the risk of long-term unemployment among those with BD to other psychiatric disorders, according to a recent study from Sweden, those with BD had a higher risk than all other disorders, when adjusted for socioeconomic factors, labor market status at baseline, and comorbid disorders (Helgesson et al., 2017). The risk was about the same for people diagnosed with OCD (Perez-Vigil et al., 2019).

Overall, during the follow-up period, over 60% of individuals diagnosed with BD experienced some form of LMM outcome, compared to 22% of the general population and 26% of their unaffected siblings. This combined measure may offer greater feasibility for comparison to previous studies that have aggregated various labor market outcomes. In doing so, our findings align with prior research indicating that approximately 40–60% of people with BD were not engaged in employment (Hakulinen et al., 2019; Marwaha et al., 2013; Sletved et al., 2021). This also echoes results from another recent register-based study from Sweden, which found that 5 years after first diagnosis, 37% of people with BD were employed (Holm et al., 2021).

When looking at the effect of sex on LMM outcomes, our findings show that women had a higher absolute risk of all LMM outcomes. However, the relative risk associated with BD for all labor market outcomes was higher for men, except for DP, where there was no difference between men and women. When considering DP, those most severely affected by BD are likely the ones in greatest need of this support and most likely to receive it, and there does not seem to be a difference between men and women regarding the severity of the disorder (Kessing, 2004). In regards to the other outcomes, it may be influenced by differences in help seeking behavior or LMM differences between men and women in the general population (Gold, 1998). Studies have shown that women are more likely to be in contact with specialist healthcare due to BD, but often have a lower intensity of service than men (Cunningham et al., 2020) and are more likely than men to be treated as outpatients rather than inpatients when first diagnosed (Kessing, 2004). Women in the general population also tend to have higher rates overall of all labor market outcomes, which likely affects the risk estimates of women with BD compared to women without BD (Gjesdal et al., 2008; Haukenes et al., 2012; Ludvigsson et al., 2019).

The risk estimates of LMM among individuals with BD were higher compared to the general population and remained higher, even when compared to unaffected siblings, although to a slightly lesser degree, showing there are severe disruptions in occupational functioning for those diagnosed with BD. The risk estimates do not decrease substantially when controlling for comorbid disorders, suggesting that BD is the main driver for LMM, not comorbid disorders, for those affected. The lower estimates in the sibling analysis suggest that there are some unmeasured confounding factors involved in work disability, particularly DP, of those with BD. Perhaps some familial factors are present, such as genetics or psychosocial factors, or experienced socioeconomic disadvantage in upbringing, that affect both the development of BD and poor labor market outcomes. In contrast, the outcome measures of SA and unemployment are more likely related to individuals who remain somewhat active in the labor market. For these individuals, differences in LMM are more likely explained by working conditions rather than by the severity of underlying health conditions or shared environment in upbringing.

### Strengths and limitations

The major strength of this study is the extensive, high-quality data available through Swedish registers that provide comprehensive information on exposure, outcome, and multiple covariates over a long period of time. Studies have shown that the validity and accuracy of diagnoses in the registers is very good (Brooke et al., 2017; Ludvigsson et al., 2011, 2016, 2019).

Another strength is the possibility to include different forms of LMM by using the registers and the large study population allowed us to control for a wide range of comorbid disorders. By examining three distinct LMM outcomes we managed to provide a more comprehensive depiction of the challenges faced by individuals affected by BD within the labor market and give a more nuanced understanding of the different ways people with BD experience LMM. Finally, by using sibling comparison, we could, to some extent, control for unmeasured factors such as genetics and environmental factors during upbringing, in a way that has not been done before when looking at LMM outcomes for people diagnosed with BD.

Some limitations include that we only have data on inpatient and specialized healthcare. Therefore, we might have missed some information on primary care visits (e.g. comorbid diagnoses in primary healthcare), that could perhaps lead to underestimation of the impact comorbid disorders have on LMM. As BD is a severe mental disorder, that would most likely be treated in specialized healthcare services, we expect minimal influence of this limitation on BD diagnosis. Another limitation is the inability to differentiate between bipolar types 1 and 2 as they share the same diagnostic codes in the ICD-10. Finally, the generalizability of the findings may be limited due to global variations in healthcare and social insurance systems.

## Conclusion

Individuals with BD face elevated risks of LMM compared to both a matched comparison group and unaffected siblings. The risk was especially pronounced in DP. Men with BD seem to have a higher relative risk of SA and unemployment than women with BD, whereas there was no difference between men and women in regards to DP.

This population-based study offers an opportunity to further our knowledge of LMM among individuals with BD. Unlike approaches focusing on employment as a binary outcome, i.e. pooling together all forms of being outside of the labor market, this study examines various outcomes, providing a more nuanced understanding of the challenges those affected with BD face in the labor market. These findings provide a potential to guide future strategies and provide additional support for policymakers and mental health services in their efforts to understand, prevent, and reduce LMM among individuals diagnosed with BD.
